# Genome-wide association analysis identifies genetic variations in subjects with myalgic encephalomyelitis/chronic fatigue syndrome

**DOI:** 10.1038/tp.2015.208

**Published:** 2016-02-09

**Authors:** K A Schlauch, S F Khaiboullina, K L De Meirleir, S Rawat, J Petereit, A A Rizvanov, N Blatt, T Mijatovic, D Kulick, A Palotás, V C Lombardi

**Affiliations:** 1 Department of Biochemistry and Molecular Biology, University of Nevada, Reno, NV, USA; 2 Nevada Center for Biomedical Research, University of Nevada, Reno, NV, USA; 3 Institute of Fundamental Medicine and Biology, Kazan Federal University, Kazan, Russian Federation; 4 R.E.D Laboratories, Zellik, Belgium; 5 Mayo Clinic, Scottsdale, AZ, USA; 6 Asklepios-Med (private medical practice and research center), Szeged, Hungary

## Abstract

Myalgic encephalomyelitis, also known as chronic fatigue syndrome or ME/CFS, is a multifactorial and debilitating disease that has an impact on over 4 million people in the United States alone. The pathogenesis of ME/CFS remains largely unknown; however, a genetic predisposition has been suggested. In the present study, we used a DNA single-nucleotide polymorphism (SNP) chip representing over 906,600 known SNPs to analyze DNA from ME/CFS subjects and healthy controls. To the best of our knowledge, this study represents the most comprehensive genome-wide association study (GWAS) of an ME/CFS cohort conducted to date. Here 442 SNPs were identified as candidates for association with ME/CFS (adjusted *P*-value<0.05). Whereas the majority of these SNPs are represented in non-coding regions of the genome, 12 SNPs were identified in the coding region of their respective gene. Among these, two candidate SNPs resulted in missense substitutions, one in a pattern recognition receptor and the other in an uncharacterized coiled-coil domain-containing protein. We also identified five SNPs that cluster in the non-coding regions of T-cell receptor loci. Further examination of these polymorphisms may help identify contributing factors to the pathophysiology of ME/CFS, as well as categorize potential targets for medical intervention strategies.

## Introduction

Myalgic encephalomyelitis, also known as chronic fatigue syndrome or ME/CFS, is a complex and heterogeneous disease that has a severe impact on the health and quality of life of those afflicted. Current estimates suggest that as many as 2.5% of adults may suffer from ME/CFS.^1, 2^ The etiology of ME/CFS is currently unknown; however, it is often characterized by neurological symptoms, memory and concentration impairment, viral reactivation and sleep abnormalities.^3^ Inflammatory sequelae and innate immune dysregulation are also commonly reported and include increased proinflammatory cytokine expression and decreased natural killer cell function and numbers, potentially leading to increased susceptibility to infection.^4, 5, 6^ In addition, ME/CFS cases often present with gastrointestinal abnormalities, which are commonly misdiagnosed initially as irritable bowel syndrome.^7, 8, 9, 10, 11^ Currently, there is no pathognomonic biomarker or clinical diagnostic test that can definitively delineate ME/CFS; therefore, diagnosis is mainly based on clinical observation and medical anamnesis with subjects meeting defined inclusion and exclusion criteria.^12, 13^

Several lines of evidence support the possibility that ME/CFS development may involve a heritable component. Albright *et al.*^14^ conducted familial clustering analysis and reported significantly higher risk for ME/CFS for first-degree relatives. In addition, studies of monozygotic and dizygotic twins suggest that there is a higher rate of fatigue concordance in monozygotic twins when compared with dizygotic twins.^15, 16^ The expression of major histocompatibility complex class II antigens HLA-DQA1*01 and HLA-DR4 has also been suggested as a potential risk factor in developing ME/CFS.^17, 18^ Moreover, single-nucleotide polymorphisms (SNPs) in the tumor necrosis factor-α and interferon-γ genes may implicate genetic factors in the dysregulation of inflammatory cytokine production.^19^

In recent years, genome-wide association studies (GWAS) have brought exciting new insights into the genetic underpinnings of many complex diseases. Polymorphisms have been identified in genes coding for proteins implicated in the disease processes of multiple sclerosis,^20^ systemic lupus erythematosus^21^ and Alzheimer's disease.^22^ In addition, GWAS have proven to be instrumental in identifying genes with complex interactions.^23, 24, 25^ Current SNP arrays allow for the concurrent screening of approximately one million known human SNPs, a capacity that has contributed notably to our knowledge of genetic influences on disease pathology.^26^ Previous to this study, only one single large (>1 00 000 SNPs) GWAS has been conducted to address the pathophysiology of ME/CFS.^27^ Smith *et al.* genotyped 40 ME/CFS subjects meeting the 1994 Fukuda *et al.* criteria^13^ and 40 controls with 1 16 204 known SNPs. Three polymorphisms of interest were highlighted: two SNPs in *GRIK2*, a gene implicated in a number of neurological maladies such as autism and schizophrenia and an SNP within the *NPAS2* gene, which is a putative circadian clock gene.

By screening ME/CFS cases and controls, we identified 442 candidate SNPs that associated with the disease cohort with statistical significance. Our pilot study supports the previous observations of other genetic screening studies and extends these results by identifying additional potential candidate polymorphisms.

## Materials and methods

### Ethics statement

This study was conducted under the guidelines of the Declaration of Helsinki. All subjects provided written informed consent before participation under a protocol approved by The University of Nevada Institutional Review Board.

### Study subjects

In this pilot study, we consented a total of 80 subjects, consistent with the previous study of Smith *et al.*:^27^ 42 cases with a confirmed diagnosis of ME/CFS and 38 healthy controls. The mean age of our disease cohort was 53.5 years (s.d. 13.0 years), and the mean age of our control cohort was 52.2 years (s.d. 8.2 years). Approximately two-thirds of our ME/CFS subjects were female (59.5%), consistent with previously reported ME/CFS demographics; approximately four-fifths of the control population were female (78.5%). All subjects identified as having ME/CFS were physician-diagnosed and met the Carruthers *et al.* criteria for myalgic encephalomyelitis as well as the 1994 Fukuda *et al.* criteria for chronic fatigue syndrome.^13, 28, 29^

### Sample size

Following the study of Smith *et al.*, that reported 64 statistically significantly associated SNPs of effect size (allelic odds ratio) ranging between 0 and 15.7, 42 cases and 38 controls were enrolled in this study. *Post hoc* power computations using QUANTO power calculator^30^ indicate that the 442 SNPs reported here have at least 80% power to detect effect sizes (allelic odds ratios) between 2.5 and 3.6, depending on minor allele frequencies (MAFs) of these SNPs. MAF values ranged from 0.08 to 0.49 in this set of 442 SNPs. QUANTO was set to compute power of allelic associations based on the additive model of MAFs between 0.08 and 0.50, significance level alpha=0.05 and population prevalence 0.003,^1, 2^ following power calculations of similar studies.^31, 32, 33^ Results from these calculations are available in the Supplementary Table 2.

### SNP analysis

Freshly isolated leukocytes were separated from whole blood using density gradient. Genomic DNA was extracted using the QIAamp DNA Mini Kit, according to the manufacturer's instructions (QIAGEN USA, Valencia, CA, USA). Aliquots of genomic DNA (250 ng) were then digested with either Nsp 1 or Sty 1. A universal adaptor oligonucleotide was then ligated to the digested DNAs. The ligated DNAs were then diluted with water and three 10-μl aliquots from each well of the Sty 1 plate and four 10-μl aliquots from each well of the Nsp 1 plate were transferred to fresh 96-well plates. PCR master mix was added to each well, and the reactions cycled as follows: 94 °C for 3 min; 30 cycles of 94 °C for 30 s, 60 °C for 45 s, 68 °C for 15 s; 68 °C for 7 min; 4 °C hold. Following PCR, the seven reactions for each sample were combined and purified using Agencourt AMPure beads (Beckman Coulter, Brea, CA, USA). The ultraviolet absorbance of the purified PCR products was measured to ensure a yield ⩾4 μg μl^−1^. Each PCR product (45 μl; ⩾180 μg) was fragmented with DNAse 1, establishing fragment sizes of less than 185 bp. The fragmented PCR products were end-labeled with a biotinylated nucleotide using terminal deoxynucleotidyl transferase.

For hybridization, the end-labeled PCR products were combined with hybridization cocktail, denatured at 95 °C for 10 min and incubated at 49 °C. Two hundred microliters of each mixture were loaded onto Affymetrix Genome-Wide SNP Array 6.0 GeneChip (Affymetrix, Santa Clara, CA, USA) and hybridized overnight at 50 °C and 60 r.p.m. Following 16–18 h of hybridization, the chips were washed and stained using the GenomeWideSNP6_450 fluidics protocol with the appropriate buffers and stains. Following washing and staining, a GeneChip Scanner 3000 was used to scan the GeneChips.

### Data analysis

The Affymetrix Genome-Wide Human SNP Array 6.0 (Affymetrix) was used to identify potential associations of 906,600 SNPs with an ME/CFS diagnosis. Affymetrix CEL files were first processed using the Corrected Robust Linear Model with maximum likelihood classification genotyping algorithm^34^ using the R package crlmm.^35^ Standard quality-control measures were used to assess the chip and sample reliability (http://www.genabel.org). Specifically, only SNPs having a MAF of at least 5% in our sample set were considered (238,664 SNPs were excluded because of having a MAF less than 5%). All SNPs were examined for low call rates (below 95%), and none were excluded because of this. A total of 3651 markers were excluded because their genotype frequencies were inconsistent with Hardy–Weinberg equilibrium (*X*^2^ raw *P*-values with *P<*0.0008). All samples studied were verified to have an SNP call rate of 95% or greater, and all sample genders were verified with heterozygosity of the X chromosome. There were 659,094 SNPs on the array passing this standard quality-control protocol across all 80 samples.

**Figure 1:**
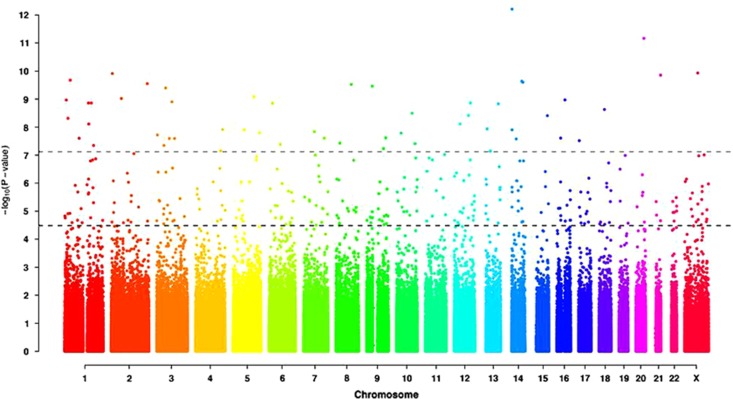
Manhattan plot of genome-wide association raw *P*-values. The black (lower) horizontal line corresponds to the false discovery rate threshold of 3.3 × 10^−5^ and the grey horizontal line corresponds to the Bonferroni threshold of 7.5 × 10^−8^.

Three single-location association tests were performed on these 6 59 094 SNPs. A genome-wide test for association was performed on each SNP via a simple logistic regression and computation of the *P*-value of the likelihood ratio test upon comparison with the null model.^36^ As the mode of inheritance is currently unknown in ME/CFS, the study was based on the codominant (additive) model, which represents the most general model available.^37^ The *P*-values of the likelihood ratio test were adjusted for multiple testing using the false discovery rate method.^38^ There were 442 SNPs with an adjusted *P*-value of *P<*0.05, corresponding to raw association *P*-values *P<*3.3 × 10^−5^. A much more stringent Bonferroni correction would target raw *P*-values in the interval (6 × 10^−16^ and 7.5 × 10^−8^). See Figure 1, in which both thresholds (false discovery rate and Bonferroni) are shown. As the NIH Catalog of Published GWAS requires statistical significance of association to be defined by raw *P*-values with *P*<1.0 × 10^−5^ (https://www.genome.gov/27529028), we highlight here the 299 statistically significant SNPs in our study with association *P*-values *P*<1.0 × 10^−5^. Note that the statistical significance threshold is not universal: for example, Smith *et al.* report SNPs with *P*-values *P*<0.01, and a similar GWAS published in 2014 reports several SNPs to be associated with Fibromyalgia at *P*-values *P*=4.28 × 10^−5^ and *P=*0.021.^39^ Thus, in Supplementary Table 1, we include 442 SNPs: the 299 SNPs with the more stringent threshold of *P*<1.0 × 10^−5^ and 143 additional SNPs with a slightly less stringent raw association *P*-value *P*<3.3 × 10^−5^.

A simple *X*^2^ hypothesis test (two degrees of freedom) for association between the three possible genotypes of each SNP and the disease trait (ME/CFS or control) was also performed on each of the 659,094 SNPs, which we refer to here as the genotypic association test. In addition, a standard Fisher's exact test (one degree of freedom) was performed on the allelic distribution between cases and controls of each SNP. *P*-values were adjusted for multiple testing using the false discovery rate method,^38^ and SNPs with an adjusted *P*-value of *P<*0.05 with respect to genotype or allelic distribution were examined carefully. For our study, this threshold corresponded to raw genotypic and allelic *P*-values in the intervals (2 × 10^−13^ and 2.2 × 10^−5^) and (8 × 10^−16^ and 2 × 10^−5^), respectively. Supplementary Table 1 includes the raw *P*-values for all three tests of association for 442 SNPs. A standard, conservative genomic control method was used to test and control for the overall inflation of the allelic association test statistic (inflation factor *λ*=1.03).^40^ The factors gender and age were considered and tested for association with ME/CFS; however, no adjustments were necessary.

SNP positions are consistent, with the 2013 human genome assembly GRCh38/hg38 (the most current major release), and are assigned to a gene if the respective SNP is within 40 kb of the gene.^41^ SNPs that are not within 40 kb of any gene are referred to as intergenic in our tables.

## Results

### Identification of SNPs that associate with ME/CFS

Of the 659,094 SNPs that passed the quality-control protocol described earlier, 407 candidate autosomal SNPs were associated with a diagnosis of ME/CFS in our cohorts (*P*<3.3 × 10^−5^; Supplementary Table 1) and 35 SNPs were identified on the X chromosome. Twenty-three SNPs were significant at *P*<1.0 × 10^−10^ (Table 1, GWAS *P*-value). The most significant SNP (rs12235235, genotypic association *P=*5.76 × 10^−16^) was identified in the intragenic region of the gene *RECK* (Reversion-Inducing Cysteine-Rich Protein With Kazal Motifs), a putative negative regulator of matrix metalloproteinases.^42^ In addition, among this group of 23 SNPs, two were in the T-cell receptor alpha locus and *TRA* (rs17255510 and rs11157573) and one in the T-cell receptor alpha/delta locus (rs10144138). We also observed an SNP in the intragenic region of the *GRIK3* gene (rs3913434), a glutamate neurotransmitter receptor and an ortholog of the *GRIK2* gene, which was identified as a highly statistically significant SNP in a previous ME/CFS GWAS conducted in 2011 by Smith *et al.* (genotypic association *P*-value *P=*0.001 and *P=*0.002).^27^

Twelve of the 442 candidate SNPs associated with the ME/CFS cohort were identified in the coding region (exon) of their respective gene: five of these were synonymous substitutions (rs16973831, rs2274515, rs3732196, rs7613828 and rs17722227), two were missense substitutions (rs2015035 and rs479448) and the remainder were within T-cell receptor or immunoglobulin loci. With respect to the missense substitutions, the observed SNP in the gene *CCDC157* (rs2015035), which codes for the coiled-coil domain-containing 157 protein, results in a non-conservative substitution of the amino acid Serine (S) to Alanine (A). The SNP in the coding region of the *CLEC4M* gene, which codes for the C-type lectin domain family 4, member M, results in a non-conservative substitution of the amino acid tyrosine (Y) to cysteine (C). Whereas the function of CCDC157 has not been fully characterized, coiled-coil domains are common motifs and function as oligomerization domains for a wide range of proteins such as structural proteins, motor proteins and transcription factors.^43^ CLEC4M, also know as L-SIGN or CD299, is a mannose-binding C-type lectin receptor, a component of the innate immune system that recognizes a broad range of pathogens.^44, 45^

### Multiple SNPs in proximity to specific genes

**Figure 2:**
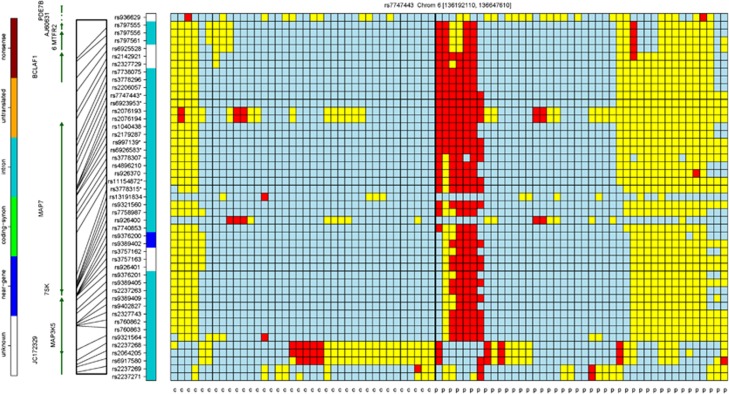
A genotypic organization of 38 controls (first 38 columns) and 42 ME/CFS cases (last 42 columns) on chromosome 6 between 136 172 610 and 136 647 610, containing the *MAP7* gene. This region contains six SNPs found to be statistically significantly associated with the ME/CFS cohort. Four of these SNPs lie in the *MAP7* region. The first seven cases show a genotypic pattern not shared by the control cohort: the red cells represent the homozygous genotype of the minor allele. The color bar directly to the left of the heatmap shows the intragenic and near-gene natures of most of the SNPs in the region. ME/CFS, myalgic encephalomyelitis/chronic fatigue syndrome.

In order to identify genotypic differences and patterns in regions near an SNP or SNPs of statistical significance, we utilized a tool specifically designed to view genotypic patterns of cases and controls simultaneously. The tool GenotypePlotter is an Open Source plotting tool, designed by co-author Schlauch, to organize both phased and unphased chromosomes in regions around potential causative SNPs of interest. This genotype-plotting tool, which is available upon request, uses a novel clustering scheme to organize samples into similar patterns based on their genotypes across a region, providing a user-friendly overview of differences between cohorts. After organizing the samples over a selection of SNPs, genotypes are portrayed in different colors to represent a type of heatmap: red cells indicate sample genotypes that are homozygous with respect to the minor allele for that SNP; blue cells indicate sample genotypes that are homozygous with respect to the major allele; and yellow cells represent heterozygous genotypes. For example, Figure 2 presents a half-megabase region containing the statistically significant SNPs rs997139, rs6926583, rs11154872, rs7747443, rs6923953 and rs3778315 on chromosome 6 in the *MAP7* gene region. This method allowed us to characterize seven ME/CFS cases that display a signature genotypic pattern across most of the regions not shared by the control cohort.

**Figure 3:**
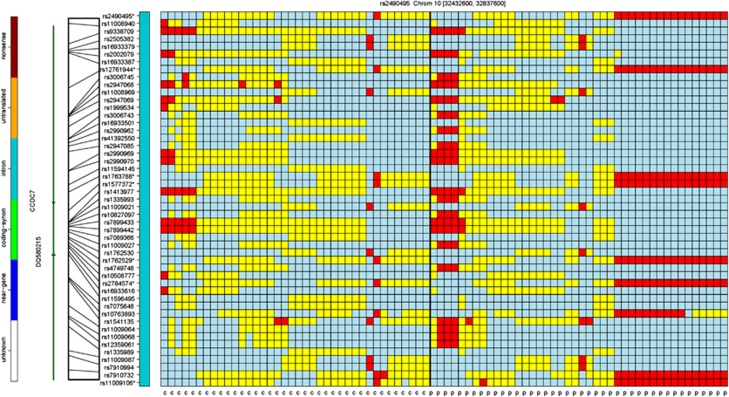
A genotypic organization of 38 controls (first 38 columns) and 42 ME/CFS cases (last 42 columns) on chromosome 10 between 32 437 590 and 32 857 600. The genotypic pattern shared by the 16 ME/CFS cases at the right of the second panel occurs in only one of the controls. ME/CFS, myalgic encephalomyelitis/chronic fatigue syndrome.

Similarly, Figure 3 shows a distinct difference between cohorts in a region on chromosome 10 that contains seven SNPs: rs2490495, rs1763788, rs1577372, rs1762529, rs2784574, rs11009106 and rs2995467, all of which lie in the *CCDC7* region. There are 16 ME/CFS cases (38%) at the far right of the image that share a distinct pattern of genotypes across the region that is shared with only one of the controls. The red cells represent the minor allele homozygous genotype of each SNP (rows). The occurrence of this type of pattern is unlikely and may represent a distinct subgroup of ME/CFS subjects.

**Figure 4:**
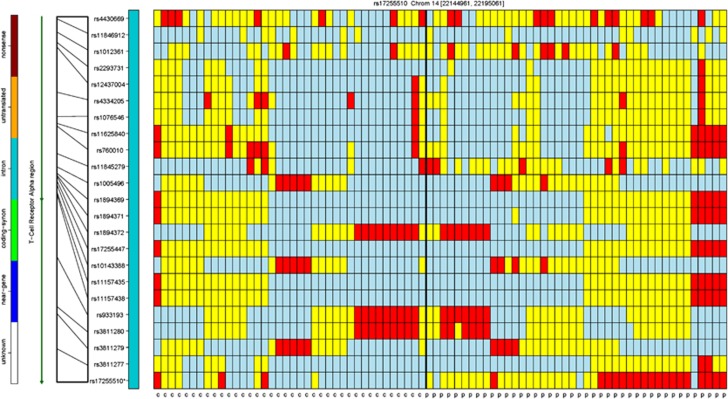
A genotypic organization of 38 controls (first 38 columns) and 42 ME/CFS cases (last 42 columns) on chromosome 14 between 22 144 961 and 22 195 061 in a region containing the *TCA* (T-cell receptor alpha) gene and the SNP rs17255510 that is statistically significantly associated with our ME/CFS cohort. There is a distinct genotypic pattern shared by the last five ME/CFS cases. ME/CFS, myalgic encephalomyelitis/chronic fatigue syndrome.

Finally, we observed three statistically significant SNPs in the T-cell receptor alpha locus and one in the T-cell receptor alpha/delta locus (rs2204978, rs17255510, rs11157573 and rs10144138, respectively), all of which occur in intragenic regions and are within half a megabase of each other on chromosome 14 (Figure 4).

To examine whether SNPs reported in the same gene are independent events, standard measures of linkage disequilibrium (LD) and *r*^2^ (correlation) are used. We report here the scaled coefficient of disequilibrium *D*' and the correlation coefficient (*r*^2^). Values of *D*'=1 indicate that two SNPs are in complete LD; *D*'=0 indicates that there is no LD between the two loci; *r*^2^=1 indicates that the two SNPs are in perfect LD. To examine whether two or more SNPs lying in the same gene have independent effects on the phenotype, we perform a simple logistic regression that includes the multiple SNPs in the model. The results of multiple SNPs reported within the *TRA*, *MAP7* and *CCDC7* are summarized here. All three SNPs reported in the *TRA* gene independently affect the phenotype. SNP pairs (rs17255510 and rs11157573) and (rs17255510 and rs10144138) are not in LD and not correlated (*D*'=0.5, *r*^2^=0.4 for both pairs), whereas the pair (rs17255510 and rs10144138) is in almost complete and perfect LD (*D*'=0.999 and *r*^2^=0.999). It is interesting to note that the length of the region in which these SNPs lie is a notable 271 kilobases.

All six SNPs reported in the *MAP7* gene have a pairwise *D*' of 0.999 or greater, and a pairwise *r*^2^ between 0.943 and 0.999. All SNP pairs have dependent effects on the CFS/ME phenotype. Note that the six SNPs lie within a relatively large region (138 kilobases). Similarly, all seven SNPs within the *CCD7* gene are in almost complete LD, with *D*' values greater than 0.974 and all pairwise *r*^2^ values greater than 0.975. These SNPs do not independently affect the genotype. Note that these seven SNPs lie in a relatively large region for LD to occur (330 kilobases).

## Discussion

Previous studies support the supposition that a genetic component is involved in ME/CFS pathogenesis. To explore this possibility, we surveyed ME/CFS cases and controls for SNPs using the Affymetrix Genome-Wide SNP Array 6.0. After initial sample-filtering, 659,054 quality SNPs were represented across all samples studied. To the best of our knowledge, this study represents the largest SNP survey of an ME/CFS cohort to date. This analysis identified 442 SNPs that reached statistical significance, and thus represent potential candidates for genetic associations with this disease. In light of the heterogeneous nature, it is possible that multiple genetic factors are involved in the pathogenesis of ME/CFS, and the results of this study may help to define specific subgroups. Indeed, previous studies have suggested that a potential genetic predisposition for immune dysregulation may exist. Using monozygotic twins to control for genetic differences, Sabath *et al.* reported that ME/CFS cases and their respective twins displayed a trend of increased circulating CD62L(+) T cells in several T-cell subsets.^46^ Other studies suggest that polymorphisms observed in subjects with ME/CFS may associate with the sleep abnormalities and the neurological dysfunction associated with this disease. Smith *et al.* utilized a convergent functional genomics approach by combining the analysis of a large-scale GWAS with an mRNA expression study to identify polymorphisms in two genes of interest in CFS subjects from the Wichita CFS Surveillance Study.^27^ Two SNPs were identified in the *GRIK2* gene, which codes for an excitatory neurotransmitter receptor that is primarily expressed in the brain. A number of neurological maladies, including autism and schizophrenia, are associated with *GRIK2*. The second identified SNP lies in the *NPAS2* gene, which is a putative circadian clock gene. Although the two SNPs identified for *GRIK2* in the Smith *et al.* study were not represented on the SNP Array 6.0, an ortholog of this gene (*GRIK3*) was observed to significantly associate with our ME/CFS cohort. Both *GRIK2* and *GRIK3* code for transmembrane subunits of neuroexcitatory receptors, belonging to the kainate family of glutamate receptors. These receptors are composed of four subunits and function as ligand-activated ion channels on presynaptic and postsynaptic neurons.

Of the 65 total SNPs identified as nominally associated with CFS (*P<*0.001) by Smith *et al.*, only 28 were represented on the current SNP Array 6.0, and, of these, only one (rs10509412, *ATAD1*) was observed to associate within our ME/CFS cohort. The mode of inheritance was not disclosed in the Smith *et al.* report, and, if different from the codominant (additive) model used in this study, it may explain why the two studies are not in agreement. In addition, given that the remaining methods used to identify SNPs are largely consistent between the two studies, it is also possible that the differences in identified SNPs reflect differences in cohorts. Subjects utilized by Smith *et al.* were derived from participants in the Wichita CFS Surveillance Study^47^ and were diagnosed with CFS according to the 1994 Fukuda *et al.* criteria.^13^ The subjects in our study were selected to meet the 2003 Canadian Case Definition of ME^28^ in addition to the Fukuda criteria. Jason *et al.* reported that, whereas the Fukuda *et al.* criteria and the Canadian Case Definition both delineate cases from chronic fatigue psychiatric controls, the Canadian criteria were more specific in selecting subjects with less psychiatric comorbidity as well as those with more physical impairment, more fatigue, more neuropsychiatric and more neurological symptoms.^48^ Notwithstanding, the identification of *GRIK*-family genes as among the most significant in both studies is consistent with a neurological component of the disease as described in the Canadian Case Definition.

Causative SNPs typically come in two forms: those located within the coding region of genes and those that reside in non-coding regions, such as the gene's regulatory sequences. In the present study, we identified 12 SNPs that occur in coding regions: two exist in immunoglobulin lambda locus; another five appear in the coding regions of genes with no functions, but are synonymous and do not alter the coding of the gene; one occurs in the open reading frame of a pseudogene; and two are missense substitutions (Supplementary Table 1). One missense substitution occurs in the *CLEC4M* gene, which codes for the C-Type Lectin Domain Family 4, Member M protein and leads to a substitution of the amino acid tyrosine (Y) to cysteine (C). The phenolic functionality of tyrosine is an important component in proteins that are part of signal transduction processes as well as acceptors of phosphate groups in kinase reactions. In contrast, cysteine, when present in pairs, can form disulfide bonds to give proteins stable secondary and tertiary structures and, individually, can serve as nucleophiles in enzymatic reactions. These two amino acids have distinct functional moieties, and therefore this polymorphism produces a non-conservative substitution and potentially may lead to decreased functionality of the receptor. *CLEC4M* is a pattern recognition receptor capable of binding to a broad range of pathogens, including hepatitis C virus,^44^ human immunodeficiency virus^45^ and *Mycobacterium tuberculosis*.^49^ A dysregulation of *CLEC4M* may have significant consequences in the pathogenesis of infectious diseases.^50^ Although ME/CFS has been associated with numerous viral infections or reactivations, including Epstein Barr virus,^51^ Enterovirus^9^ and Parvovirus B19,^52^ a causative infectious agent has never been identified. Future studies will be required to determine whether a *CLEC4M* polymorphism may predispose subjects with ME/CFS to viral infection.

The other missense substitution occurs in the *CCDC157* gene, which codes for the poorly characterized Coiled-Coil Domain Containing 157 protein. RNA-Seq analysis has identified CCDC157 transcripts in many tissues including the brain, the small intestine and the kidney (http://www.gtexportal.org/home/gene/CCDC157), suggesting that this protein of unknown function likely has an important physiological role. In addition, the coiled-coil motif is important in many biological processes such as the regulation of gene expression (transcription factors); however, a greater understanding of this protein will be required before its potential role in ME/CFS may be fully considered.

Whereas 12 SNPs were observed in coding regions of their respective genes, over 96% of the significant SNPs identified in our study occur in non-coding regions. However, it is well documented that SNPs residing within introns, or those upstream or downstream of genes, also have the capacity to be causal.^53, 54, 55, 56^ In fact, in a recent study, Farh *et al.* utilized a fine-mapping algorithm to analyze GWAS data for 21 autoimmune diseases and reported that ~90% of all causal variants map to non-coding regions.^57^ They further reported that only 10–20% of causal SNPs directly alter recognizable transcription factor-binding motifs. These observations suggest that SNPs within proximity of a given gene need to be considered in the context of the gene function as well as the disease phenotype.

We also identified three regions with multiple statistically significant SNPs in proximity to specific genes. One region includes four statistically significant SNPs in the *MAP7* gene on chromosome 6; another interesting area contains seven statistically significant SNPs in the region of the *CCDC7* gene on chromosome 10 and another four within T-cell receptor loci. Our knowledge of *MAP7* and *CCDC7* is very limited at this time. *MAP7* is a retinoic acid-inducible gene primarily expressed in cells of epithelial origin.^58^ Overexpression of *CCDC7* has been associated with a number of malignancies;^59, 60^ however, to the best of our knowledge, a polymorphism in this gene has not been associated with any disease. The probability of several SNPs being found to be statistically significant in a relatively small region is remote. However, the identification of multiple SNPs within a single gene that associated with disease is not without precedence. For example, multiple SNPs within the *RNASEL* gene have been associated with prostate cancer,^61^ and several SNPs in the *CDKN2B* and *ANRIL* genes have been associated with cardiovascular disease.^62^ In addition, the distinct haplotypic patterns of some of the cases in these regions may suggest that these genes are involved in a mechanism that separates the ME/CFS cases into distinct subgroups. However, given the lack of knowledge of these genes, their involvement in disease association is not obvious and requires further investigation.

The implications of multiple SNPs in the intragenic regions of *TCA* loci are more obvious. In the thymus, the *TCA* gene undergoes somatic recombination to give rise to diverse amino-acid sequences in the antigen-binding regions of the alpha chain of T-cell receptors. T-cell receptors recognize antigens bound to major histocompatibility complex class I and class II molecules and, therefore, are critical components of adaptive immunity. Major histocompatibility complex can present antigens from nearly all forms of pathogens; however, T cells that recognize 'self' antigens and have escaped negative selection in the thymus can promote autoimmune disease. Indeed, polymorphisms in the *TCA* locus have been described in association with autoimmune disease.^63^ For example, based on familial associations with the human leukocyte antigen allele DQB1*0602, an autoimmune etiology had long been suggested for the sleep disorder narcolepsy; however, the identification of a polymorphism in the *TCA* locus provided the convincing evidence.^64^ If the association of these SNPs in the *TCA* locus is confirmed in a larger ME/CFS cohort, this observation may also provide evidence of an autoimmune component in this disease.

In summary, the data presented in this study, to the best of our knowledge, represent the largest SNP survey in an ME/CFS cohort. The strengths of the study presented here include the notably dense and comprehensive genome-wide coverage: this study tested the possible association of more than 650,000 good quality known human SNPs with ME/CFS. In addition, the ME/CFS cohort was selected with great care and detail adhering to both the Carruthers *et al.* criteria for myalgic encephalomyelitis as well as the 1994 Fukuda *et al.* criteria for chronic fatigue syndrome. Although the study is limited in its sample size, the stringency of the quality control protocol and the statistical significance threshold of SNPs reported in this study adheres to typical standards of GWAS.

Using an ultra-high-density SNP genotyping array, we screened cases and controls to identify 442 potential loci that associate with ME/CFS. Previous studies support the contention that a genetic component may have a role in ME/CFS pathophysiology. Hence, the SNPs identified and reported here may help direct future research efforts in a more specific manner by identifying biological pathways and genes that may be associated with disease progression.

## Figures and Tables

**Table 1:** Twenty-three most significant SNPs based on the GWAS and genotypic association test *P*-value

*Chrom*	*Posn*	*SNP ID*	*Gene*	*Genotype*				P*-value*	
					*Ctrl*	*CFS*	*GWAS*	*Genotypic test*	*Allelic test*
1	36983994	rs3913434	*GRIK3*	CC	37	11	1.26E−11	7.15E−10	1.06E−09
				CT	1	30			
				TT	0	1			
2	7643373	rs270838	*LOC101929510*	AA	30	3	3.61E−11	5.72E−10	2.84E−07
				AC	8	38			
				CC	0	1			
	65650464	rs6757577	*KRT18P33*	GG	33	7	2.77E−10	2.74E−09	3.00E−08
				AG	5	33			
				AA	0	2			
	231342446	rs16827966	*ARMC9*	CC	37	12	5.32E−11	2.84E−10	6.24E−09
				CT	1	30			
3	56871895	rs6445832	*ARHGEF3*	AA	32	6	4.36E−10	3.99E−10	2.84E−07
				AG	6	36			
	97300204	rs1523773	*EPHA6*	AA	38	15	4.73E−11	1.26E−09	2.68E−09
				AT	0	27			
5	135086514	rs254577	*C5orf66*	CC	3	25	2.35E−11	4.42E−09	8.22E−12
				CT	14	17			
				TT	21	0			
6	22141516	rs41378447	*CASC14*	CC	32	4	1.06E−11	1.72E−10	2.61E−09
				CT	5	32			
				TT	1	6			
8	96338727	rs7010471	*PTDSS1*	AA	34	8	2.49E−10	2.99E−10	6.93E−08
				AG	4	34			
9	36091136	rs12235235	*RECK*	CC	34	3	5.76E−16	1.84E−13	2.08E−08
				CT	2	38			
				TT	2	1			
	119856753	rs7849492	*—*	TT	28	3	9.95E−10	8.13E−09	1.78E−06
				CT	9	36			
				CC	1	3			
12	91754952	rs12312259	*—*	TT	26	2	3.60E−10	9.30E−09	2.48E−07
				CT	12	34			
				CC	0	6			
13	99394905	rs9585049	*UBAC2*	AA	35	10	5.25E−10	6.06E−09	2.85E−08
				AT	3	31			
				TT	0	1			
14	22194962	rs17255510	*TRA*	TT	28	3	6.61E−10	6.29E−09	6.70E−11
				CT	7	21			
				CC	3	18			
	22420786	rs11157573	*TRA*	AA	29	4	2.97E−10	2.85E−09	9.81E−06
				AG	6	35			
				GG	3	3			
	22464970	rs10144138	*TRA/TRD*	CC	36	6	6.99E−14	6.21E−13	2.91E−10
				CT	2	36			
	84743518	rs17120254	*—*	AA	11	42	5.20E−13	1.65E−10	4.70E−12
				AT	24	0			
				TT	3	0			
	91917655	rs2249954	*FBLN5*	AA	32	5	5.47E−11	7.14E−10	4.86E−08
				AG	6	35			
				GG	0	2			
15	91945362	rs8029503	*SLCO3A1*	CC	31	4	5.66E−11	6.70E−10	1.28E−07
				CT	6	35			
				TT	1	3			
16	52532950	rs3095598	*TOX3*	TT	35	9	1.02E−10	1.73E−09	2.25E−09
				CT	3	30			
				CC	0	3			
18	37241025	rs948440	*CELF4*	TT	28	3	3.92E−10	5.76E−09	2.81E−07
				CT	10	35			
				CC	0	4			
20	52341088	rs41493945	*—*	GG	37	9	6.25E−13	6.82E−12	4.27E−10
				AG	1	33			
21	43928298	rs3788079	*AGPAT3*	AA	38	13	3.42E−12	1.40E−10	4.82E−10
				AC	0	29			

Abbreviations: Chrom, chromosome; Ctrl, control; GWAS, genome-wide association study; Posn, position; SNP, single-nucleotide polymorphism.
