# Supplementary material for: Genome-wide association analysis identifies genetic variations in subjects with myalgic encephalomyelitis/chronic fatigue syndrome
Source: Transl Psychiatry. 2016 Feb 9;6(2):e730–. doi: 10.1038/tp.2015.208 (PMC4872418; doi:10.1038/tp.2015.208)
Supplement: Supplementary Information [file tp2015208x1.pdf]

Outcome:	Disease
Design:	Unmatched case-control (1:1)
Hypothesis:	Gene only
Desired power:	0.800000
Significance:	0.050000, 2-sided
Gene	
Mode of inheritance:	Log-additive
Allele frequency:	0.0800 to 0.5000 by 0.0700
Disease model	Summary parameters
*P_0 0.003000	k_P 0.003000
R_G: 1.0000	(*indicates calculated value)

Parameter	Null	Full	Reduced
Gene	β_G=0	β_G	——

		N	
Frequency	R_G	Gene	P_0
0.080000	1.0000	Cannot calculate	0.003000
	1.5000	551	0.002775
	2.0000	170	0.002574
	2.5000	90	0.002395
	3.0000	59	0.002234
	3.5000	44	0.002090
	4.0000	34	0.001959
	4.5000	28	0.001841
	5.0000	24	0.001733
0.150000	1.0000	Cannot calculate	0.003000
	1.5000	329	0.002597
	2.0000	104	0.002271
	2.5000	57	0.002004
	3.0000	38	0.001781
	3.5000	29	0.001594
	4.0000	23	0.001435
	4.5000	20	0.001299
	5.0000	17	0.001182
0.220000	1.0000	Cannot calculate	0.003000
	1.5000	252	0.002437
	2.0000	82	0.002019
	2.5000	46	0.001701
	3.0000	32	0.001452
	3.5000	24	0.001255
	4.0000	20	0.001095
	4.5000	17	0.000965
	5.0000	15	0.000856
0.290000	1.0000	Cannot calculate	0.003000
	1.5000	217	0.002290
	2.0000	72	0.001806
	2.5000	41	0.001461
	3.0000	29	0.001207
	3.5000	22	0.001013
	4.0000	19	0.000863
	4.5000	16	0.000744
	5.0000	14	0.000648

0.360000	1.0000	Cannot calculate	0.003000
	1.5000	200	0.002157
	2.0000	68	0.001625
	2.5000	40	0.001269
	3.0000	28	0.001018
	3.5000	22	0.000835
	4.0000	18	0.000697
	4.5000	16	0.000591
	5.0000	14	0.000508
0.430000	1.0000	Cannot calculate	0.003000
	1.5000	193	0.002035
	2.0000	67	0.001470
	2.5000	40	0.001112
	3.0000	28	0.000871
	3.5000	23	0.000700
	4.0000	19	0.000575
	4.5000	17	0.000481
	5.0000	15	0.000408
0.500000	1.0000	Cannot calculate	0.003000
	1.5000	195	0.001923
	2.0000	69	0.001336
	2.5000	41	0.000983
	3.0000	30	0.000753
	3.5000	24	0.000595
	4.0000	20	0.000482
	4.5000	18	0.000399
	5.0000	16	0.000335

N is the number of cases required for the desired power
The required number of controls is 1xN
